# Thermovision: a new diagnostic method for orofacial pain?

**DOI:** 10.2147/JPR.S183096

**Published:** 2018-12-13

**Authors:** Jitka Fricova, Marketa Janatova, Martin Anders, Jakub Albrecht, Richard Rokyta

**Affiliations:** 1 Charles University, 1st Faculty of Medicine, General University Hospital, Department of Anesthesiology, Resuscitation and Intensive Medicine, Pain Management Center, Prague, Czech Republic, j.fricova@seznam.cz; 2 Charles University, 3rd Faculty of Medicine, Department of Normal, Pathological and Clinical Physiology, Prague, Czech Republic, j.fricova@seznam.cz, richard.rokyta@lf3.cuni.cz; 3 Charles University, 1st Faculty of Medicine, General University Hospital, Department of Rehabilitation Medicine, Prague, Czech Republic; 4 Charles University, 1st Faculty of Medicine, General University Hospital, Department of Psychiatry, Prague, Czech Republic

**Keywords:** orofacial pain, thermovision, infrared thermography, transcranial stimulation

## Abstract

**Background:**

Infrared thermography can be used to obtain more complete information about a patient’s condition. The method can be used in various medical applications for monitoring acute and chronic orofacial pain syndrome. With this diagnostic method, thermal differences in the examined region are usually compared to the same reference region on the opposite side of the body.

**Methods:**

Infrared quantitative thermography is a non-invasive method for contactless monitoring of dynamic thermal fields on a surface, or in this case, the human body. This method is based on detection of infrared radiation, which is naturally emitted from the surface of the body. In a pilot project with a patient having orofacial pain, changes before and after repetitive transcranial magnetic brain stimulation treatment were assessed.

**Results:**

First-day measurements found significantly higher maximum, minimum, and average temperatures, before and after therapy, in the area where the patient subjectively reported pain. The fifth and final measurements, before and after therapy, found only a slight elevation of the maximum temperature of the assessed regions, relative to the same regions on the opposite side of the face.

**Conclusion:**

During the measurements on the fifth day, a thermal difference greater than 0.4°C was only observed relative to the minimum temperatures associated with the regions of self-reported pain before and after therapy. For validation of the effects, this method will need to be tested using a randomized, double-blind study with a larger number of patients.

## Introduction

Objective measurement of pain intensity is a continuing and difficult problem in pain management, which is particularly demanding for certain types of pain, such as orofacial pain. In this paper we present our original idea for assessing pain intensity using thermal imaging. Pain was assessed using thermal imaging before and after each therapy session, which consisted of non-invasive repetitive transcranial magnetic stimulation (rTMS). The prevalence of orofacial pain varies significantly. Depending on the study, it affects 10%–50% of the adult population. Orofacial pain is often accompanied by a history of sore gums or teeth. Orofacial pain can appear after dental treatment or dental surgery. Atypical odontalgia,^1^ which is a frequent diagnosis for orofacial pain, is very often pharmacoresistant. Currently there are no established criteria for what should be considered pharmacoresistance with regard to neuropathic pain. If efforts are to be made toward solving the problem of patients who report lack of significant pain relief despite having tried multiple drug monotherapy, a definition of pharmacoresistant neuropathic pain is critical.^2^ Atypical odontalgia is described as persistent idiopathic pain that does not meet the diagnostic criteria for cranial neuralgias and cannot be attributed to another disorder.^3^ It is often “throbbing” and/or “burning” in nature.^4^ Chronic facial pain can be localized on one side or both sides with a permanent or intermittent course. New research suggests involvement of the peripheral and central nervous system as a possible mechanism for atypical odontalgia pathophysiology.^5^ Diagnostic criteria for orofacial pain can be found in the recommended guidelines of the International Association for the Study of Pain^6^ and in the guidelines of the International Headache Society 2013.^7^ Nonetheless, there are differences between these guidelines, and few clinical studies dealing with orofacial pain diagnoses have been performed.^8^ From a clinical point of view, the clearest method is to divide chronic orofacial pain based on time (episodic or continuous) and location (ie, one side or both sides). It is also possible to use the more conventional classification, which is based on the cause of the pain, ie, pain can be divided into neuropathic or vessel pain. From a diagnostic point of view, it is crucial to recognize that facial pain can be secondary to carcinoma or metastasis. Many patients with orofacial pain are placed under the diagnosis “atypical orofacial pain”, where an unambiguous cause has not been found. In such cases, terminology can be slightly misleading, since the diagnosis is often used because a better one cannot be found. For instance, secondary trigeminal neuralgia and atypical odontalgia are usually placed under this diagnosis. Over the past 10 years, the number of patients with orofacial pain, after corrective dental surgeries, has increased. A new study found that somatosensory abnormalities were evident in atypical odontalgia and inflammatory dental pulpitis patients. Somatosensory changes were still present in dental pulpitis patients 3 months after pulpectomy. However, no somatosensory changes were found after implant placement.^9^ In chronic pain there is a crucial relationship between brain activity and normal and abnormal pain sensations,^10^ which can be affected by rTMS. Lack of clinically relevant somatotopic effects in upper limb or face pain suggests that much of the rTMS analgesic effect may depend on high-order mechanisms involving cognitive and affective appraisal of pain, rather than on a sensory effect related to the specific motor area stimulated.^11^ It remains to be determined whether the interest of theta burst stimulation (TBS) priming is to generate a simple additive effect or a more specific process of cortical plasticity.^12^ Not only patients with chronic pain but also patients with Alzheimer’s disease can benefit from rTMS procedure, in terms of cognitive performances, apathy, and dependence, even in the long term. These promising results remain to be confirmed in controlled studies based on a larger population size, which could also help identify the prognostic factors associated with good outcome, in order to optimize patient selection.^13^ Chronic orofacial pain is very often associated with other symptoms (apathy, depression), and sometimes we also diagnose various comorbidities in these patients. Comorbidity between fibromyalgia and migraine involves heightened somatic hyperalgesia compared to one condition only.^14^

### Current methods for orofacial pain diagnosis

With this diagnostic method, thermal differences in the examined region are usually compared to the same reference region on the opposite side of the body. For an accurate diagnosis, it is essential to get a comprehensive medical history, which includes sufficient time to allow the patients to complete their opening statement. As with all chronic pain, a pain history should include: a psychological assessment, family history (eg, temporomandibular disorders [TMDs] have a genetic predisposition), social history, and significant life events. It is also useful to determine which health care professionals the patients have consulted for their problem, including complementary and alternative medicine practitioners. A full pharmacologic history is important as well as a past and present medical history.^15^ Red flags include giant cell arthritis, which must be distinguished from TMDs, especially in those >50 years old, and cancer, which can present as progressive neuropathic pain.^16^ The large US OPPERA study confirmed the complexity and showed that TMD is not just isolated facial pain.^17^ It is crucial to define all pain conditions as precisely and rigidly as possible in order to ensure a homogenous population. This ensures the least variability when rating the pain, which will consequently allow for combining and comparing research, on a particular population, across different professional settings. This is not easy for chronic facial pain because there is a lack of verifiable morphological causes or structural lesions, and because these symptoms are often rather featureless. The new International Association for the Study of Pain classification of chronic pain is a big step toward better characterization of such conditions and should stimulate future work on a new and operationalized classification of orofacial pain.^18^

### Thermal imaging: a new diagnostic method in pain management?

The fact that many patients with orofacial pain mention dental treatment or dental surgery in their medical history and the pain appeared to be associated with inflammation, led us to focus on inflammation as a potential diagnostic feature. The existence of inflammation seems to be associated with orofacial pain. Since common blood tests and imaging methods have failed to verify or identify the origin of inflammation, we decided to test if thermal imaging could be used to identify an inflammatory heat signature. A change in temperature, in the affected region, is one of the main characteristics of inflammation. Therefore, thermal imaging can be used to identify and localize inflammation.

### Infrared quantitative thermography

Infrared quantitative thermography is a non-invasive method for contactless monitoring of dynamic thermal fields on a surface, or in this case the human body. This method is based on detection of infrared radiation, which is naturally emitted from the surface of the body. The emission of radiation is related to surface temperature. Emissivity of human skin is 0.96°C–0.98°C (0= perfect reflector and 1= perfect emitter).^19^ The radiation is converted into an electric signal, which is then transformed into a thermal image illustrating the spatial distribution of superficial temperatures. Monitoring body temperature as a diagnostic tool of a patient’s condition was used as early as the fourth century BC. The first thermometer was invented in the 17th century. Modern infrared quantitative thermography started being used in medicine in the 1960s, and thanks to technological progress, the method became much more common in the 1990s.^19^,^20^ Thermography is normally used as an auxiliary diagnostic tool both in clinical practice and in research. The use of this method enables specialists to obtain more complete information about a patient’s condition. When using this method, it is necessary to use standardized procedures and conditions. The patient must be properly informed regarding the necessity to follow the therapeutic regimen as well as all thermal imaging protocols in order to avoid distortion of examination results. The temperature in the room must be stable. The optimum ambient temperature during measurements needs to be 18°C–25°C, depending on the diagnostic region and disease.^21^

#### Infrared quantitative thermography in medical applications

Thermography is used in various medical applications, eg, diagnosis of inflammatory diseases, reflex sympathetic dystrophy syndrome, internal injuries, and tumor diseases.^19^,^22^–^25^ It can also be used to monitor implant healing, demarcation of burns and frostbite, vascular dysfunctions, and skin diseases.^26^,^27^ In ophthalmology, thermography is used to detect changes in the thermal field of the eyes and for monitoring corneal temperature during an operation.^28^ Thermography can similarly be used to monitor physiotherapy.^29^ When monitoring acute and chronic pain syndromes in the orofacial region, thermography can be used eg, to help diagnose odontalgia, sinusitis, temporomandibular joint disorders, and idiopathic trigeminal neuralgia.^30^ Using an analysis of thermal fields, information regarding the course of pathologies associated with an increase or decrease of skin temperature can be obtained. Inflammatory processes (hyper-perfusion) manifest on thermograms as areas with elevated temperature, while hypo-perfusion shows up as areas with lower temperatures. Therefore, we can, with high specificity and sensitivity, detect eg, changes in the vasospastic reaction to cold (using cold water) in patients with Raynaud phenomenon or disorders of vascular adaptation resulting from abnormal autonomous nervous system function, or delayed reactions to thermal changes in patients with complex regional pain syndrome.^31^,^32^ The duration and reduction of inflammation have been objectively monitored, by studies focused on the effects of corticosteroids on the treatment of rheumatoid arthritis, by observing thermal changes on the skin surface over inflamed joints.^33^ A correlation between thermographic examinations and the severity of knee osteoarthritis, diagnosed using skiagraphy, has also been established.^34^ It is always necessary to assess which thermal deviations are pathological. In muscle spasms, elbow bursitis, tendovaginitis, fibromyalgia, and in acute muscle injuries, elevated temperature are detected, while in chronic tissue damage, scars, and paretic muscles, lower temperatures are measured.^19^ Thermography is also a sensitive method for detection and localization of skin thermal changes of nervous origin.^35^ When assessing the effect of therapy, it is important to remember the potential of a placebo effect in patients with chronic neuropathic pain.^36^ Skin temperature is influenced by blood circulation, which is controlled by the autonomous nervous system. Central control mechanisms of skin temperature, under physiological conditions, affect both sides of the body evenly. This presents as a symmetry of thermal gradients around the body’s central axis. An analysis of thermal pictures can reveal changes in thermal distribution patterns and differences between similar areas on the right and left side of the body.

## Methods

### Thermal imaging in orofacial pain

**Figure 1:**
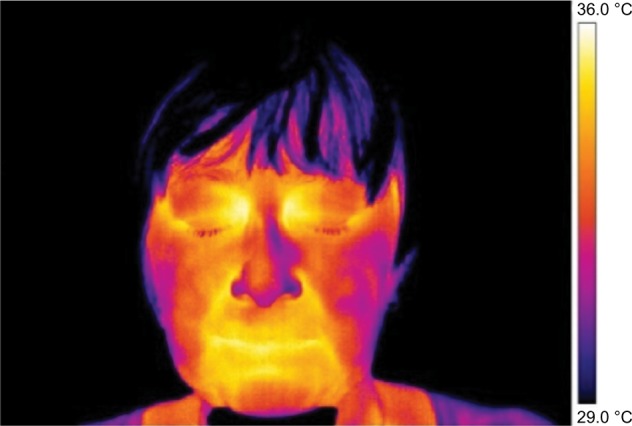
Thermography of orofacial area before therapy (orofacial pain – right side). **Note:** This figure shows that asymmetry in the thermal fields on the right and left side of the nose and cheek is evident.

**Figure 2:**
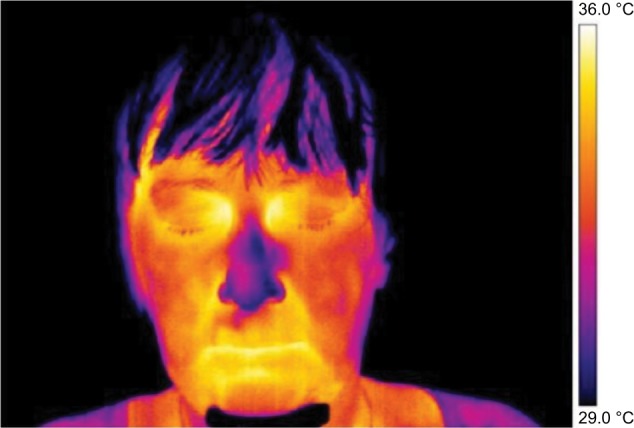
Thermography of orofacial area after first therapy session (contralateral rTMS was applied). **Note:** This figure shows that asymmetry in the thermal fields on the right and left side of the nose and cheek is evident. **Abbreviation:** rTMS, repetitive transcranial magnetic stimulation.

**Figure 3:**
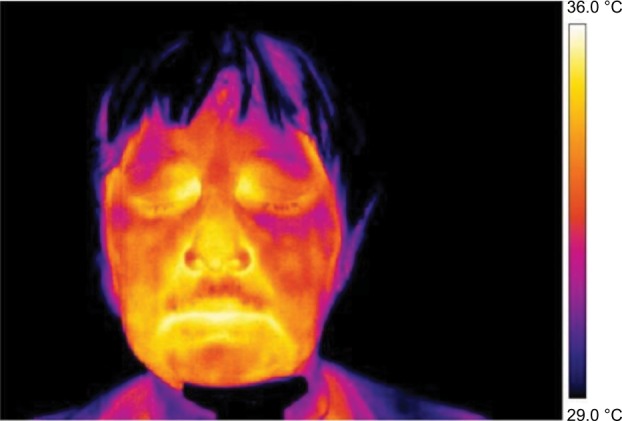
Thermography of orofacial area before fifth (final) therapy session.

**Figure 4:**
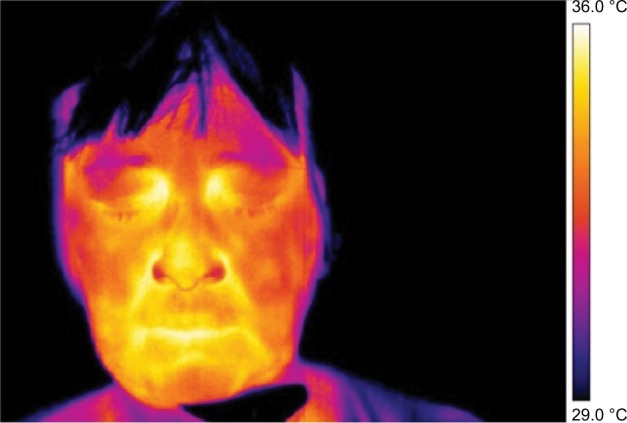
Thermography of orofacial area after fifth (final) therapy session.

**Figure 5:**
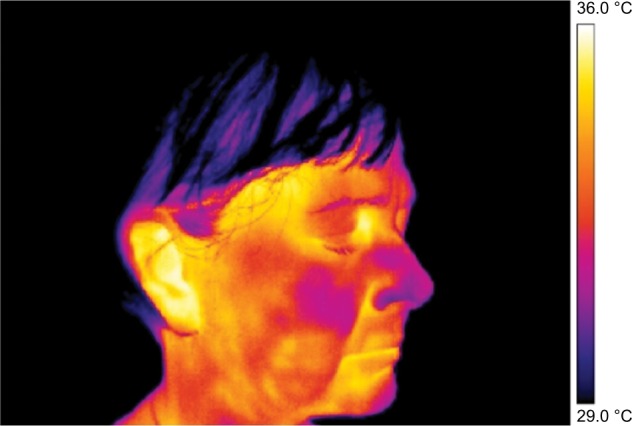
Thermography (detailed) of right cheek after rTMS therapy. **Abbreviation:** rTMS, repetitive transcranial magnetic stimulation.

**Figure 6:**
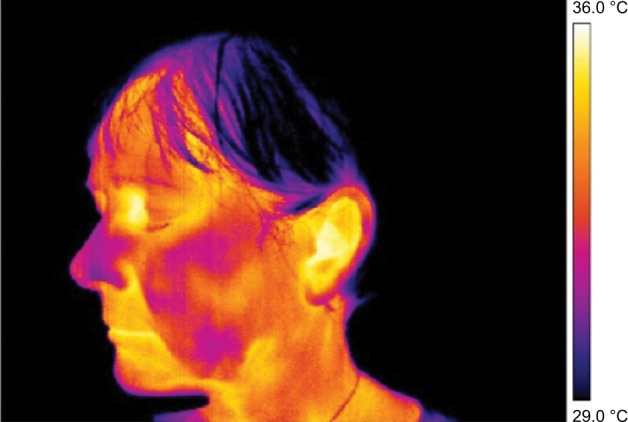
Thermography (detailed) of left cheek after rTMS therapy. **Abbreviation:** rTMS, repetitive transcranial magnetic stimulation.

All procedures performed in this study involving a human participant were in accordance with the ethical standards of the institutional and/or national research committee and with the Declaration of Helsinki. Approval from the local Ethics Committee of General University Hospital in Prague was obtained. Informed consent regarding publication was obtained from the patient included in the study. The patient signed an informed written consent to enter the research project, the informed consent was approved by the local and central Ethics Committee. For this study, the thermal imaging was conducted in the Department of Psychiatry, in a room with stable temperature and humidity. Repeated thermographic images were taken of a patient with orofacial pain who underwent 5 days of therapy using rTMS. A ThermaCAM^™^ (ThermaCAM^™^ E300; FLIR^®^ Systems, Inc., Wilsonville, OR, USA) thermal camera was used for thermal measurements. The system was equipped with high sensitivity microbolometric detectors with a resolution of 320×240 pixels, a frequency up to 50 Hz, and a sensitivity of 0.1°C. Thermal camera imaging was always performed under the same technical conditions, ie, the same place, room temperature (23.4°C–23.7°C), humidity (59.9%–62.4%), and atmospheric pressure (996–998 kPa). There was minimal airflow and no direct sunlight on the patient. The thermal camera was placed on a stand and adjusted so that no reflective surfaces were present in the image. There were no external heat sources in the room. The patient’s face was not covered. Thermal imaging was done 1 hour before treatment and again 1 hour after treatment. The patient was tested after sitting in a quiet room for 20 minutes. The room temperature was between 20°C and 24°C. All the previously mentioned guidelines were followed, the patient was informed, in advance, about the procedure protocol. The patient was instructed to not drink any alcohol during the 12 hours before testing. The patient did not eat, drink, or use a cell phone an hour before the examination. Additionally, the patient agreed to forgo acupuncture or manipulative treatment for at least 72 hours prior to the examination. On the day of the examination, the skin was dry and clean without any use of cosmetic products. Medications were kept unchanged across all measurements. One week before the rTMS analgesic medication was stable. Before the beginning of each measurement, the patient localized and self-assessed the pain, which was recorded. During the course of the measurement, the patient was seated in an armchair used for thermographic examinations. Positioning and distance between the thermal camera and the region of the patient’s face under examination were individualized for the patient. A rotating arm was modified so that the thermal camera could be optimally adjusted in all imaging directions. For precise positioning of the required angle, a protractor fastened above the diagnostic armchair was used. Imaging was performed the first and fifth day before and after each treatment session. Measurements were always taken at a constant distance and directly oriented toward the examined region. Three pictures were taken of each side of the face: a frontal and lateral view, and from an angle of 45°. Researcher Pro software system was used to evaluate the thermal images. This software makes it possible to correct and analyze thermal data. Point, line, surface, and other analyses offer quantitative assessments. The results are graphic images (Figures 1–6). The affected regions, ie, the shape and size of the thermal field, were identified in these images. The main focus was on the differences between the assessed and reference part of the opposite side of the face. Two types of regions were compared in the assessment: the region demarcated by thermal fields and the region in which the patient subjectively reported pain. Differences between thermal maximum, minimum, and average on both the right and left sides were compared. A thermal difference greater than 0.4°C between the left and right side of the face was considered meaningful.

### rTMs

rTMS is a method that is able to influence not only the pain but also other symptoms accompanying pain. Application of rTMS was directed toward the contralateral motor cortex associated with the painful area described by the patient. Stimulation was performed using a Magstim Rapid^2^ (Magstim, Whitland, Carmarthenshire, UK). The Magstim Rapid^2^ is a system capable of high frequency rTMS. TBS was used since it is capable of producing long-term and effective changes in the stimulated cortex after a relatively short (lasting tens of seconds (20–190 seconds) application, from a 70 mm double air film coil.

## Results

The Researcher Pro software system was used to evaluate the thermal images. This software makes it possible to correct and analyze thermal data. Point, line, surface, and other analyses offer quantitative assessments. The results are graphic images shown in Figures 1–6.

Differences in measured temperature between duplicate pictures from the same region and on the same side of the face were on the order of 0.1°C±0.1°C.

Frontal pictures were chosen for the assessment of thermal differences between symmetrical facial parts (Figures 1–4).

In Figures 3 and 4 the thermal fields on the left and right side are symmetrical, although, slight asymmetry in the temperature of the cheeks persists.

In the lateral view images, asymmetry in thermal distribution is evident. However, it did not correspond to the self-reported pain location on which rTMS therapy had been focused (Figures 5 and 6).

On the first day of rTMS therapy the patient reported a pain grade of 6, based on the pain intensity scale. Subjective localization was on the right side of the nose spreading to the whole cheek. On the second and third day the patient reported a pain grade of 2 in the same region. On the fourth and fifth day the patient reported an increase in pain.

**Figure 7:**
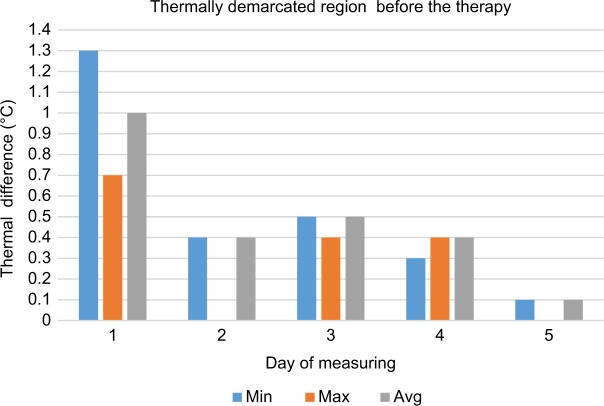
Thermal differences of thermally demarcated regions before therapy. **Abbreviations:** avg, average; max, maximum; min, minimum.

**Figure 8:**
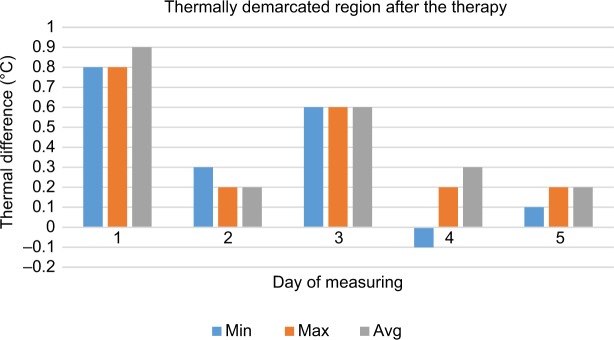
Thermal differences of thermally demarcated regions after therapy. **Abbreviations:** avg, average; max, maximum; min, minimum.

The pain on the right side of the nose and right cheek was assessed as a subjective sore spot (highly localized). However, under thermal imaging, the region of the lesion (inflamed region) was large with significant asymmetry with an increased temperature focused on the right side of the nose. During our analysis of the thermographs, results from the same regions taken on the fifth day were compared with those taken on the first day. The area of asymmetrical thermal increase on the nose, seen on the first day, was found to be not significantly warmer than the reference area on the opposite side of the face, on the fifth day (Table 1). During the first measurements, there was a significantly more elevated temperature seen in the regions of the self-reported pain and in the region demarcated by the thermal fields compared to the reference areas on the opposite side of the face. This difference was seen in the minimum, maximum, and average temperature before and after the first treatment session. Before and after the third treatment session, a significant difference in the thermally demarcated areas (ie, minimum, maximum, and average temperature) was evident. Nevertheless, a difference in self-reported pain was not obvious. There was a significant difference in the minimum, maximum, and average temperature of the thermally demarcated regions between the before and after images from the first therapy session and the after images from the third therapy session (Figures 7 and 8). During the measurements on the fifth day, a thermal difference greater than 0.4°C was only observed relative to the minimum temperatures associated with the regions of self-reported pain before and after therapy.

## Discussion

It is always necessary to determine which thermal deviations are signs of pathology. In muscle spasms, elbow bursitis, tendovaginitis, fibromyalgia, and acute muscle injuries, for example, an elevated temperature is observed, while in chronic stage tissue damage, scars, and paretic muscles, skin surfaces are hypothermic.^19^,^21^ From our results it is possible to conclude that thermovision is a sensitive method for locating thermal changes associated with pain in the skin. When assessing the effect of a therapy, using thermography, it is important to remember that in patients with chronic neuropathic pain the placebo effect may, in part, explain the observed results.^36^ Skin temperature is influenced by blood circulation, which is in turn, controlled by the autonomous nervous system. Central control mechanisms regulating skin temperature, under physiological conditions, affect both sides of the body evenly, which presents as thermal symmetry along the central axis. In an analysis of thermal images, differences in thermal distribution and changes between the corresponding right and left sides of the body are monitored. Selfe et al considered a thermal difference of 0.5°C to be significant when trying to distinguish between a healthy and arthritic knee.^38^ Other painful conditions of the joints, which are associated with inflammation, will probably not be diagnosed with this method. Wilson et al, in setting criteria for a complex regional pain syndrome, used a thermal difference ≥1°C (between the left and right sides).^39^ Gratt et al found that the maximum thermal difference between symmetrical facial areas, in a group of 102 healthy asymptomatic participants, was 0.4°C±0.1°C.^37^ In a study with 164 patients with orofacial pain, a difference >0.35°C was considered significant. In a control group of healthy probands the thermal difference between symmetrical facial parts was 0.1°C±0.1°C. In a majority of patients with orofacial pain of various origins, an increase or decrease in skin temperature of the affected region relative to the opposite symmetrical side was found to be ≥0.4°C.^37^

## Conclusion

This case study demonstrates the usefulness of thermal imaging in localizing inflammation associated with orofacial pain. In this pilot project, the use of infrared thermography, as an auxiliary aid in the diagnosis of orofacial pain, was demonstrated. In our patient we found significantly elevated average, maximum, and minimum temperatures in the facial region of the self-reported pain compared to the corresponding region on the opposite side of the face. In the final measurement, ie, after 5 consecutive day rTMS treatments, what had been pronounced thermal asymmetry between the right and left sides of the face was gone. The patient also reported reduced pain in the area. Despite its high accuracy and sensitivity, infrared thermography should be viewed as an auxiliary method and could be complementary to comprehensive investigation. To verify the value of this method, it is necessary to take other results into consideration and carry out a more elaborate randomized, double-blind study with a larger number of patients. Thermal imaging seems to be a promising, as well as inexpensive tool that can be used to visualize inflammation associated with or perhaps causing orofacial pain. We are aware of the limitations of the thermovision method in the diagnosis of orofacial pain and also the need to continue to study and specify the methodology of the study.

## Figures and Tables

**Table 1:** Differences in temperature between symmetrical facial parts before and after rTMS treatment

Measuring	Region	Difference of the minimum (°C)	Difference of the maximum (°C)	Difference of the average (°C)
Before first therapy session	Thermally demarcated	**1.3**	**0.7**	**1**
Before first therapy session	Self-assessed	**1.5**	**0.6**	**0.8**
After first therapy session	Thermally demarcated	**0.8**	**0.7**	**0.9**
After first therapy session	Self-assessed	**0.8**	**0.5**	**0.8**
Before second therapy session	Thermally demarcated	0.4	0	0.4
Before second therapy session	Self-assessed	0.1	0.2	0.2
After second therapy session	Thermally demarcated	0.3	0.2	0.2
After second therapy session	Self-assessed	0.3	0	0.2
Before third therapy session	Thermally demarcated	**0.5**	0.4	**0.5**
Before third therapy session	Self-assessed	0.3	0	0.2
After third therapy session	Thermally demarcated	**0.6**	**0.6**	**0.6**
After third therapy session	Self-assessed	−0.2	−0.2	−0.2
Before fourth therapy session	Thermally demarcated	0.3	0.4	0.4
Before fourth therapy session	Self-assessed	0.4	0	0.3
After fourth therapy session	Thermally demarcated	−0.1	0.2	0.3
After fourth therapy session	Self-assessed	**0.6**	0.3	0.1
Before fifth therapy session	Thermally demarcated	0.1	0	0.1
Before fifth therapy session	Self-assessed	**0.8**	0.2	0.4
After fifth therapy session	Thermally demarcated	0.1	0.2	0.2
After fifth therapy session	Self-assessed	**0.5**	0.3	0.3

**Note:** Significant values of 0.5°C–1.5°C are shown in bold.

**Abbreviation:** rTMS, repetitive transcranial magnetic stimulation.
